# Correlated random walks caused by dynamical wavefunction collapse

**DOI:** 10.1038/srep13380

**Published:** 2015-08-25

**Authors:** D. J. Bedingham, H. Ulbricht

**Affiliations:** 1 Faculty of Philosophy, University of Oxford, OX2 6GG, United Kingdom; 2 School of Physics and Astronomy, University of Southampton, SO17 1BJ, United Kingdom

## Abstract

Wavefunction collapse models modify Schrödinger’s equation so that it describes the collapse of a superposition of macroscopically distinguishable states as a dynamical process. This provides a basis for the resolution of the quantum measurement problem. An additional generic consequence of the collapse mechanism is that it causes particles to exhibit a tiny random diffusive motion. Here it is shown that for the continuous spontaneous localization (CSL) model—one of the most well developed collapse models—the diffusions of two sufficiently nearby particles are positively correlated. An experimental test of this effect is proposed in which random displacements of pairs of free nanoparticles are measured after they have been simultaneously released from nearby traps. The experiment must be carried out at sufficiently low temperature and pressure in order for the collapse effects to dominate over the ambient environmental noise. It is argued that these constraints can be satisfied by current technologies for a large region of the viable parameter space of the CSL model. The effect disappears as the separation between particles exceeds the CSL length scale. The test therefore provides a means of bounding this length scale.

Dynamical wavefunction collapse models^1^,^2^ provide a unified description of quantum dynamics encompassing both unitary evolution and state reduction. Typically the standard Schrödinger dynamics are extended so that the state behaves stochastically and in such a way that certain superposition states are unstable. The most prominent collapse model is the continuous spontaneous localization (CSL) model^3^,^4^ in which a superposition of quasi-localized matter states will collapse at a rate which increases with the mass of the object. This results in the rapid collapse of macroscopically distinguishable superposition states whilst micro states are little affected.

Since the CSL model involves a modification of the Schrödinger equation, it makes predictions which are in conflict with standard quantum theory. It is therefore possible to experimentally test CSL. A direct way to do this is to try to observe quantum interference for objects of increasing mass. Detailed studies show that there should be a characteristic loss of fringe visibility as the mass increases beyond a sufficient size (see Refs 5, 6, 7). Another observable effect of CSL is a random diffusive motion of particles. An isolated object will undergo a random walk induced by the collapse mechanism. As shown in Ref. 8 this can dominate over environmental effects at sufficiently low temperature and pressure.

Here we consider a situation with two non-interacting particles. We will examine how the particles behave as a result of the CSL dynamics and show that there is a potentially measurable effect whereby the diffusions undergone by each particle are correlated. A key feature of the CSL model is that the localization mechanism acts on the total smeared mass density state rather than individually on each particle. This suggests that for two nearby particles, the diffusive behaviour caused by the localization mechanism will be correlated. This should be apparent in the joint spatial probability distribution. In what follows we shall solve the two-particle CSL master equation to determine the behaviour precisely.

Our proposed experiment is shown schematically in Fig. 1. A pair of nanoparticles are trapped side by side using laser light. The particles are simultaneously released and allowed a brief period of free fall. The positions of each of the particles is then measured by light (Rayleigh) scattering. The experiment does not involve matter wave interferometry and therefore avoids much technical complication. Indeed the scheme can be performed with existing technology.

We assume that the two particles are non-interacting and of equal mass. We also assume that the two particles are identical bosons although this is not crucial to our argument—the same conclusion holds if the particles are non-identical. We could also regard the particles as composite objects made up of many constituent particles satisfying the CSL dynamics. This case is more complicated but it can be shown^4^ that the CSL dynamics can be applied to the composite object as a whole. Furthermore, any environmental particles involved in the CSL dynamics but not part of the system of interest can be traced away without effect.

There are two fixed parameters in the CSL model which are treated as fundamental constants. These are the localization rate *λ* and the localization length scale 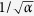. We consider a regime in which the localization length scale is large compared to the length scale defining the spatial extent of the system. The original estimates of these parameters by Ghirardi, Rimini and Weber (GRW)^9^ are *λ* = 10^−16^ s^−1^ and 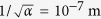. However, these values are not definitive. A recent study of the valid regions of the *λ*-*α* parameter space consistent with experiment puts no upper bound limit on the CSL length scale^10^.

The CSL master equation is given by


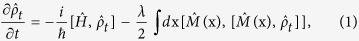


with density operator 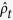 and free Hamiltonian 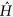. The smeared mass density operator is given by


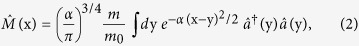


where *m*_0_ is the nucleon mass (used to set the mass scale), and the field annihilation and creation operators 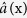 and 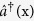 satisfy


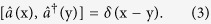


The two-particle state is represented by


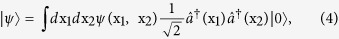


where 1 and 2 label the two particles and *ψ* is a symmetric wavefunction. In this representation improper position eigenstates are given by


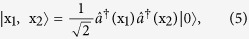


and the two-particle density matrix is represented in coordinate space as


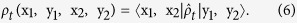


The coordinate space representation of Eq. (1) for the two-particle state in the limit of large localization length is found to be


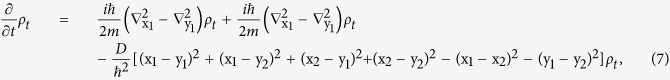


where


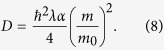


Here, the limit of large localization length specifically means that *ρ*_*t*_(x_1_, y_1_, x_2_, y_2_) is only significant whenever 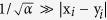 for *i* = 1, 2 and 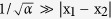 (see Refs 11, 12).

Equation (7) applies in three dimensional space. In what follows we are concerned only with the behaviour in one dimension. We choose this to be the horizontal dimension of Fig. 1. We can trace over the coordinates of the other spatial dimensions in *ρ*_*t*_ resulting in a reduced one dimensional density matrix. It is straightforward to show that this satisfies a master equation with the same form as Eq. (7).

We now present a solution of Eq. (7) (further details of the techniques used can be found in Ref. 12). The solution is represented in terms of a density matrix propagator *J* (see Refs 13, 14) as


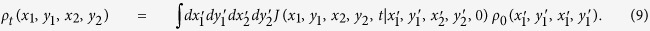


We find that for Eq. (7) (in one dimension), *J* is given by


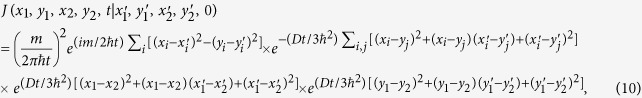


where *i*, *j* = 1, 2.

We consider an initial wave function of the form


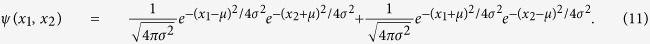


This represents the particles residing in adjacent harmonic traps of width *σ* and spaced by a distance 2*μ* ≫ *σ*. The initial density matrix is


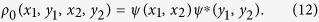


We suppose that the particles are simultaneously released from the traps and allowed to diffuse freely. However, they remain sufficiently separated that there is negligible conventional interaction between them. Using Eqs (9 and 10) we have calculated the diagonal part of the density matrix


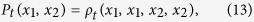


following a time period *t* after their release. This represents the joint probability distribution for the subsequently measured positions of the two particles. The result is simplest when expressed in terms of the variables


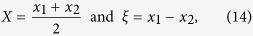


where we find


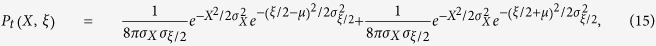


with


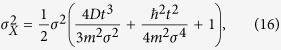



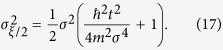


The distribution consists of two peaks, one about *X* = 0, *ξ*/2 = *μ* and another about *X* = 0, *ξ*/2 = −*μ*. Note that we have ignored a possible interference term between the two peaks in Eq. (15) since we assume that they do not disperse enough to overlap. The feature that we are interested in is the shape of these peaks and in particular their rate of dispersion in the two directions *X* and *ξ*/2. The *X* and *ξ* spreads of each of the peaks are defined by *σ*_*X*_ and *σ*_*ξ*/2_ respectively. We see that the CSL parameter *D* contributes to *σ*_*X*_ whilst *σ*_*ξ*/2_ is unaffected. Note that by setting *D* = 0 (standard quantum mechanics) in Eqs (16 and 17) we find 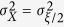. This reflects the fact that the two particles are behaving independently and their distributions are uncorrelated.

The physical reason for the difference between *σ*_*X*_ and *σ*_*ξ*/2_ when *D* ≠ 0 is that the diffusive shifts in position of the two particles caused by the collapse mechanism will be positively correlated if the particles are closer together than the localization length scale. The system as a whole diffuses but *ξ* = *x*_2_ − *x*_1_ is only affected by standard quantum dispersion.

Our result is clearly dependent on the fact that the localization length scale 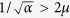, but in this limit the spreads in *X* and *ξ*/2 do not depend on the separation 2 *μ*. For 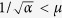 the particles would behave independently under CSL. A null observation would therefore put a constraint on *α*.

To observe the effect we must distinguish 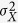 and 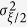. We can estimate these variances by repeatedly measuring the displacements of two particles simultaneously dropped from nearby traps (see Fig. 1). We assume that the traps are such that *σ *= 10 nm and the particles are released for *t* = 0.25 s corresponding to a drop of 30 cm. We further assume that position measurements have an error of *σ*_*err*_ = 10 nm which is normally distributed and independent of *x*_*i*_. If 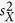 is the unbiased estimate of 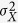 based on *n* sets of position measurements, then the variance in 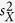 is given by


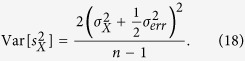


To be able to clearly observe the effect of CSL we demand that 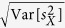 should be at least 10 times smaller than the difference


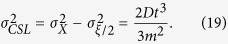


Taking the mass of the particles to be 10^9^ amu such that *ħ*^2^*t*^2^/4*m*^2^*σ*^4^ ≪ 1, this results in the following constraint on *n*:


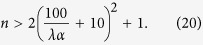


This is shown in Fig. 2(a).

Now we consider the effects of an environment. We suppose that the particles are confined within a vacuum chamber and consider the constraints placed on temperature and pressure by the condition that the collapse effects should be dominant. We consider environmental noise contributions both from radiation and molecular collisions.

It is shown in Ref. 15 that the dominant contribution to the thermal diffusion by radiation is due to recoil from emission of photons. There it is also shown that the variance in displacement of a bulk sphere of radius *R* and density 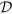 due to the emission of radiation is given (in SI units) by


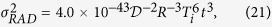


where *T*_*i*_ is the internal temperature of the bulk object. By demanding that 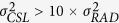 we can constrain the internal temperature of the particles. Using 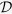 = 10^3^ kgm^−3^ and *R* = 10^−7^ m, we find


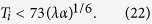


The maximum internal temperature is shown in Fig. 2(b).

The mean time between molecule-sphere collisions in the impact regime is given in seconds by^8^


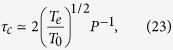


where *P* is the pressure given in picoTorr, *T*_*e*_ is the external temperature, and *T*_0_ = 300 K is room temperature. We make the conservative assumption that *τ*_*c*_ should be 10 times greater than the total experiment time *n* × *t*. Taking *T*_*e*_/*T*_0_ ∼ 1 this results in


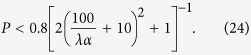


The maximum ambient pressure in the vacuum chamber is shown in Fig. 2(c).

We conclude that an experiment can be performed to test for *λα* as low as 1 m^−2^s^−1^. This requires *n* = 24000; *T*_*i*_ = 73 K and *P* = 3.3 × 10 ^− 17^ Torr. We assume a separation between traps of at least 1 mm (to avoid dispersion forces/gravity effects) so that the correlation effect should be present for 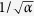 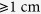. The accessible region of *λ*-*α* parameter space is shown in Fig. 3. Also shown is the region of parameter space currently ruled out by diffraction experiments involving particles of mass 10^5^ amu^16^.

In summary, we have demonstrated an effect of the CSL model in which the diffusive behaviour of two sufficiently nearby particles is correlated. If one particle is found to have randomly moved in one direction as a result of the collapse mechanism, the other particle is more likely to have moved in the same direction. We propose attempting to observe this effect as a test of CSL against standard quantum theory and as a specific test of the CSL length scale. The experiment is possible with today’s technology.

## Additional Information

**How to cite this article**: Bedingham, D.J. and Ulbricht, H. Correlated random walks caused by dynamical wavefunction collapse. *Sci. Rep.* **5**, 13380; doi: 10.1038/srep13380 (2015).

## Figures and Tables

**Figure 1:**
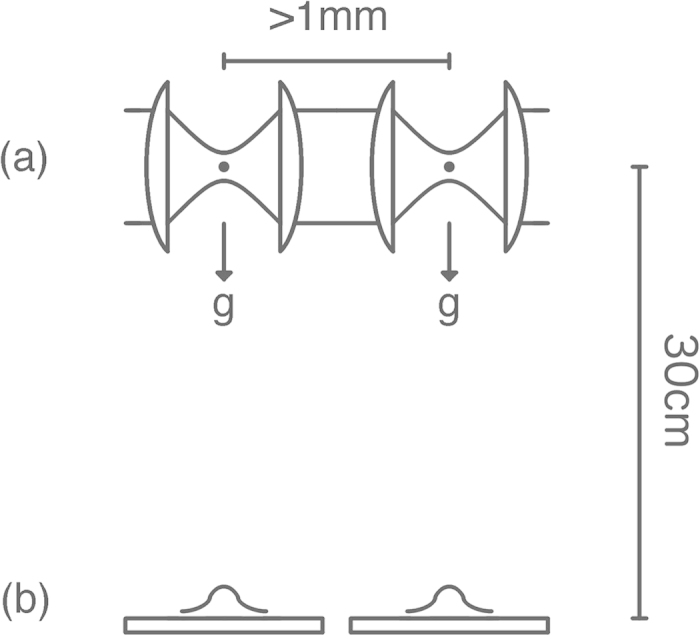
The experimental setup: (**a**) Laser light creates adjacent harmonic traps for two nanoparticles separated by of order 1mm. The particles are simultaneously released and fall 30 cm. During this period the wave packets of the two particles undergo quantum dispersion. A dynamical wavefunction collapse mechanism will also cause diffusion of the centre of mass of each of the wave packets. The diffusions of the two packets will be correlated if the localization length scale is greater than the particle separation. The position of each particle is recorded with 10 nm accuracy at (**b**).

**Figure 2:**
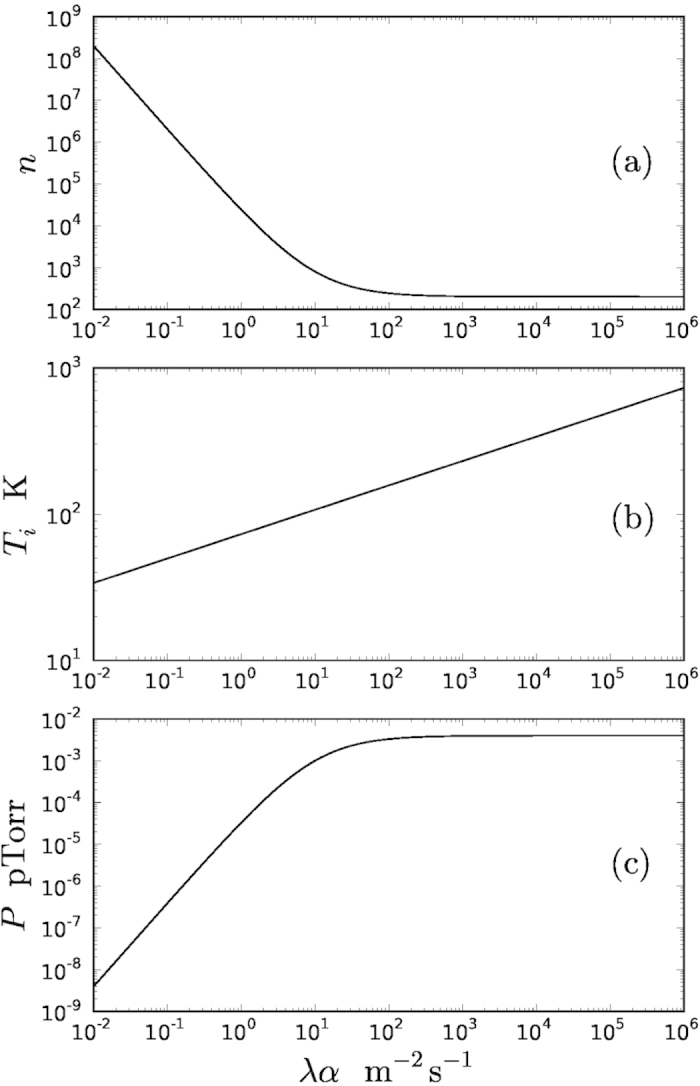
(**a**) Minimum value for the number of observations *n* required to estimate the variance 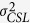 with sufficient accuracy. The statistical error in the estimate should be an order of magnitude less than 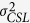. (**b**) Maximum internal temperature *T*_*i*_ of particles required to prevent significant thermal diffusion by emission of radiation. (**c**) Maximum ambient pressure *P* required to prevent significant diffusion due to collisions between ambient molecules and the nanoparticles of interest. In cases (**b**) and (**c**) the boundary is determined by the requirement that the environmental contributions to the variance of the displacement of the nanoparticle should be less than 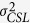 by an order of magnitude.

**Figure 3:**
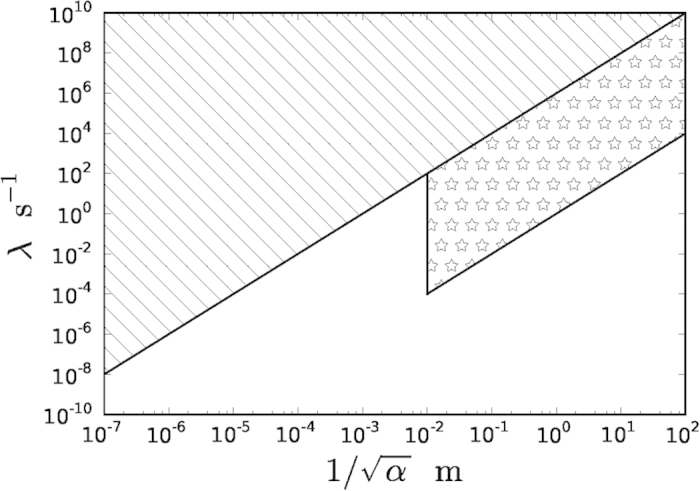
CSL parameter diagram. The hatched area shows the region of *λ*-*α* parameter space currently ruled out by diffraction experiments. The starred area shows the region of parameter space in which the correlated random walks are experimentally accessible.

## References

[b1] Bassi. A. & Ghirardi G. C. Dynamical reduction models. Phys. Rept. 379, 257–426 (2003).

[b2] Bassi A., Lochan K., Satin S., Singh T. P. & Ulbricht H. Models of wave-function collapse, underlying theories, and experimental tests. Rev. Mod. Phys. 85, 471–527 (2013).

[b3] Pearle P. Combining stochastic dynamical state-vector reduction with spontaneous localisation. Phys. Rev. A 39, 2277–2289 (1989). 9901493 10.1103/physreva.39.2277

[b4] Ghirardi G. C., Pearle P. & Rimini A. Markov process in Hilbert space and continuous spontaneous localisation of systems of identical particles. Phys. Rev. A 42, 78 (1990). 9903779 10.1103/physreva.42.78

[b5] Nimmrichter S., Hornberger K., Haslinger P. & Arndt M. Testing spontaneous localisations theories with matter-wave interferometer. Phys. Rev. A 83, 043621 (2011).

[b6] Marshall W., Simon C., Penrose R. & Bouwmeester D. Towards quantum superpositions of a mirror. Phys. Rev. Lett. 91, 130401 (2003). 14525288 10.1103/PhysRevLett.91.130401

[b7] Kippenberg T. & Vahala K. Cavity optomechanics: back-action at the mesoscale. Science 321, 1172 (2008). 18755966 10.1126/science.1156032

[b8] Collett B. & Pearle P. Wavefunction collapse and random walk. Found. Phys. 33 1495 (2003).

[b9] Ghirardi G. C., Rimini A. & Weber T. Unified dynamics for microscopic and macroscopic systems Phys. Rev. D 34, 470 (1986). 10.1103/physrevd.34.470 9957165

[b10] Feldmann W. & Tumulka R., Parameters diagrams of the GRW and CSL theories of wavefunctions collapse. J. Phys. A: Math. Theor. 45, 06504 (2012).

[b11] Bedingham D. J. Single Particle energy diffusion from relativistic spontaneous localisation. Phys. Rev. D 88, 045032 (2013).

[b12] Bedingham D. J. Effects of the continuous spontaneous localization model in the regime of large localisation length. Phys. Rev. A 89, 032713 (2014).

[b13] Caldeira A. O. & Leggett A. J. Path integral approach to quantum Brownian motion. Physica A 121, 587 (1983).

[b14] Anastopoulos C. & Halliwell J. J. Generalized uncertainty relations and long-time limits for quantum Brownian motion models. Phys. Rev. D 51, 6870 (1995). 10.1103/physrevd.51.6870 10018450

[b15] Bera S., Motwani B., Singh T. P. & Ulbricht H. A proposal for the experimental detection of CSL induced random walk. Sci. Rep. 5, 7664: 10.1038/srep07664 (2015). 25563619 PMC4288224

[b16] Eibenberger S., Gerlich S., Arndt M., Mayor M. & Tüxen J. Matter-wave interference of particles selected from a molecular library with masses exceeding 10000 amu. Phys. Chem. Chem. Phys. 15, 14696 (2013). 23900710 10.1039/c3cp51500a

